# Response of human dental pulp cells to a silver-containing PLGA/TCP-nanofabric as a potential antibacterial regenerative pulp-capping material

**DOI:** 10.1186/s12903-017-0348-7

**Published:** 2017-02-27

**Authors:** Barbara Cvikl, Samuel C. Hess, Richard J. Miron, Hermann Agis, Dieter Bosshardt, Thomas Attin, Patrick R. Schmidlin, Adrian Lussi

**Affiliations:** 1 Department of Preventive, Restorative and Pediatric Dentistry, School of Dentistry, University of Bern, Bern, Switzerland; 2 Institute for Chemical and Bioengineering, Department of Chemistry and Applied Biosciences, ETH Zurich, Zurich, Switzerland; 3 Department of Conservative Dentistry & Periodontology, Medical University of Vienna, Vienna, Austria; 4 Robert K. Schenk Laboratory of Oral Histology, Department of Periodontology, Department of Oral Surgery and Stomatology, University of Bern, Bern, Switzerland; 5 Clinic of Preventive Dentistry, Periodontology and Cariology, Center of Dental Medicine, University of Zurich, Plattenstrasse 11, Zurich, CH-8032 Switzerland; 6 Department of Periodontology, College of Dental Medicine, Nova Southeastern University, Fort Lauderdale, Florida, USA

**Keywords:** Dental pulp, Regeneration, Capping, In vitro techniques

## Abstract

**Background:**

Damage or exposure of the dental pulp requires immediate therapeutic intervention.

**Methods:**

This study assessed the biocompatibility of a silver-containing PLGA/TCP-nanofabric scaffold (PLGA/Ag-TCP) in two in vitro models, i.e. the material adapted on pre-cultured cells and cells directly cultured on the material, respectively. Collagen saffolds with and without hyaluronan acid (Coll-HA; Coll) using both cell culturing methods and cells growing on culture plates served as reference. Cell viability and proliferation were assessed after 24, 48, and 72 h based on formazan formation and BrdU incorporation. Scaffolds were harvested. Gene expression of interleukin(IL)-6, tumor necrosis factor (TNF)-alpha, and alkaline phosphatase (AP) was assessed 24 h after stimulation.

**Results:**

In both models formazan formation and BrdU incorporation was reduced by PLGA/Ag-TCP on dental pulp cells, while no significant reduction was found in cells with Coll and Coll-HA. Cells with PLGA/Ag-TCP for 72 h showed similar relative BrdU incorporation than cells stimulated with Coll and Coll-HA. A prominent increase in the pro-inflammatory genes IL-6 and TNF-α was observed when cells were cultured with PLGA/Ag-TCP compared to the other groups. This increase was parallel with a slight increase in AP expression. Overall, no differences between the two culture methods were observed.

**Conclusions:**

PLGA/Ag-TCP decreased viability and proliferation rate of human dental pulp cells and increased the pro-inflammatory capacity and alkaline phosphatase expression. Whether these cellular responses observed in vitro translate into pulp regeneration in vivo will be assessed in further studies.

## Background

The dental pulp represents the vital core of teeth and lays largely hidden under a protective shell, which forms the teeth, i.e. dentin, cementum and enamel [1]. The cells physiologically constitute together with the lining layer of odontoblasts to the cellular aspect of the pulp-dentin complex, which allows for several complex adaptive and reactive processes during all stages of tooth development and life [2, 3]. Several factors may lead to gene up- or down-regulation or even to cause cell death–in the worst-case. In the latter scenario, odontoblastoid cells have to differentiate first from the subodontoblast layer or have to be recruited from specific progenitor cells from the pulp core. The formation of reactionary or reparative dentin is the result of these self protecting reactions during injury [4, 5]. Reparative dentin forms at a highly variable degree [6] depending on the specific physical or chemical influences. Irrespective of the pathological agent, dentinogenesis would be a desirable therapeutic endpoint of healing or even regeneration. But in many cases, exposure of the pulp requires immediate therapeutic intervention as it mostly relates to maceration of this highly vulnerable and susceptible organ. In addition, any pathological changes including microbial contamination have probably preceded and the pulp-dentin complex has been infected. Therefore, agents applied in such cases should exhibit both, antibacterial and mineralizing activity. The mineralizing activity may be – classically – achieved by some irritating action of the selected material or induction of release of biologically active molecule or a combination thereof [7].

As the development of mineralized tissues in the human body, i.e. bone, cementum or dentin is based on calcification processes within a fiber arrangement, the use of modern scaffolds in the form of fibers may potentially offer some advantages, especially if these materials are doped with particles that show distinct antibacterial and mineralizing characteristics. In this context, biodegradable polymers, such as poly(lactic acid) (PLA) and the co-polymerpoly(lactic-co-glycolic acid) (PLGA) have been widely investigated and applied to fabricate porous scaffolds in order to restore damaged tissues [8]. A flexible, moldable, electrospun cotton wool-like nanocomposite has been developed [9–11], which contains amorphous calcium phosphate nanoparticles embedded in a biodegradable synthetic PLGA. It is prepared through an electrospinning process, which gives it the typical cotton wool-like appearance. The incorporation of inorganic substances and organic substances within composite scaffolds has been shown to enhance biomineralization. In addition, L-poly(lactic acid) and PLGA composite scaffolds, especially when combined with basic substances like hydroxyapatite, tricalcium phosphate or demineralized bone powder, have also shown not to induce inflammatory tissue reactions in vivo, thus seem to be highly biocompatible [12]. These materials can be optionally doped with silver, which has shown to result in enhanced antimicrobial properties against *Escherichia coli* when compared to tetracycline controls [13]. Furthermore, a preclinical study in sheep showed indirect evidence that–besides its extraordinary in vitro bioactivity – a silver containing nanocomposite material (0.4 wt.% total silver concentration) could provide additional antimicrobial properties for treating bone defects exposed to microorganisms and the absence of clusters of lymphocytes, plasma cells and spindle type fibroblasts further confirmed its biocompatibility [14].

Type I collagen from dentinal tissue on the other hand has also the ability to self-assemble and to form repetitive structures and extended networks of aligned fibers [15]. Its interactions with non-collagenous proteins [16] and its positional relationship with mineral crystals within mature tissues [17] provide convincing evidence that the preassembled collagen matrix could serve as a template for organize d mineralization in dentin.

It was the aim of this in vitro study to assess the response of human dental pulp cells to a novel silver-containing nanofabric (PLGA/Ag-TCP) and compare it to collagen (Coll) scaffolds with or without the addition of hyaluronan acid (HA). The cell viability, proliferation, and expression of the pro-inflammatory genes interleukin(IL)-6, tumor necrosis factor (TNF)-alpha as well as alkaline phosphatase (AP) expression were assessed in two in vitro models: Cells were cultured in presence of the scaffold (Model I), which might more closely represent the in vivo situation of a pulp capping and the cells were cultured directly on the scaffold (Model II).

We hypothesized that PLGA/Ag-TCP induces minor cellular responses with regards to viability, proliferation, and pro-inflammatory capacity when compared to Coll scaffolds with and without hyaluronan acid, which is known in the research field of wound healing [18] and that the differences are more pronounced in the model I than in model II.

## Methods

### Scaffolds and materials

For the fabrication of the silver-containing nanofabric, clinically approved poly(lactide-co-glycolide) (PLGA) with a copolymer ratio of 85:15 (Resomer Sample MD Type RG) was purchased from Boehringer Ingelheim with a weight and number average molecular weight of 380,300 and 181,900 g mol^−1^, respectively. Fibers with PLGA/Ag-TCP containing 2% silver in a 70:30 ratio were fabricated by an electrospinning process [13]. Each electrospinning solution was prepared with a concentration of 5.9% (w/w) PLGA in chloroform (Riedel de Haen, Ph. Eur.) containing 6.4% (w/w) of the surfactant Tween20 (Polysorbate20, Fluka, Ph. Eur.) referred to the polymer. For the preparation of the electrospinning solution, corresponding amounts of the nanoparticles were first dispersed in a chloroform/Tween20 stock solution using an ultrasonic processor at 70 W for 5 min. PLGA was subsequently added and dissolved for 15 h by magnetic stirring. Electrospinning was performed by feeding the solutions through four capillaries (inner diameter 1.0 mm) using a syringe pump. The feeding rate was set to 8 mL h^−1^. A voltage of 24 kV was applied to the needle tips, which was kept in a chloroform–air stream (160 mlmin^−1^) by a concentrically mounted polyetheretherketone (PEEK) adapter. A positively charged jet was formed from the Taylor cone and was sprayed onto a rotating (100 rpm) collection tube covered by an aluminium foil. The distance between the needle tip and the collection tube (diameter 8 cm) was kept constant at 17 cm. The as-spun scaffolds were dried and stored in vacuum at room temperature.

As reference, a collagen scaffold with and without hyaluronan acid (Coll-HA, Coll) was used in the present study (Bio-Gide^®^, Geistlich, Wolhusen, Switzerland; LOT 81500056; molecular weight of collagen 1 300 KDa). The hyaluronan acid (Regedent, Zurich, Switzerland; LOT MK2-1714/1) applied on the membrane contained 2 mg sodium hyaluronate (molecular weight 2.5 MDa), 16 mg cross-linked sodium hyaluronate (molecular weight 1 MDa), 6.9 mg sodium chloride and water for injection ad 1 ml (Vol. 1.2 ml).

### Cell culture

Human dental pulps of two anonymous donors below 30 years were isolated from caries-free third molars after informed consent was obtained (Ethics Committee of the Medical University Vienna; EK 631/2007). Dental pulp tissues were left in Dulbecco’s Modified Eagle Medium (DMEM, Invitrogen Corporation, Carlsbad, CA, USA) supplemented with 10% fetal bovine serum (FCS; PAA Laboratories, Linz, Austria) and antibiotics (Invitrogen) at 37 °C, 5% CO_2,_ and 95% humidity. Dental pulp cells grown out from the pulp were cultured. Cells that had not undergone more than five passages were used and cell seeding for indicated experiments was performed in growth medium at 30,000 cells/cm^2^. The cell density was based on previous in vitro studies on the impact of biomaterials on cell activity [19]. Experiments on viability and proliferation were performed with two different cell donors, each in duplicates. Gene expression analyses were performed twice, each time in duplicates, using pooled dental pulp cells of the two donors.

### Preparation and pretreatment of the scaffolds

For MTT tests and BrdU incorporation assays silver-containing nanofabric (PLGA/Ag-TCP) scaffolds and collagen (Coll) scaffolds of Ø 6 mm were gained from corresponding scaffolds using a biopsy punch. For gene expression, analysis scaffolds of Ø 2.2 cm were cut. One half of the collagen scaffolds was soaked in cross-linked hyaluronan acid for 1 min and thoroughly washed with PBS for 2 min (Coll-HA) immediately before stimulation of the cells.

### In vitro models (Model I, Model II)

Two different in vitro models were used. Cells were precultured and the scaffold was placed thereon (Model I – scaffold on cells) and cells were cultured directly on the scaffold (Model II cells on scaffold). In both models cell viability and proliferation was assessed based on formazan formation (MTT test) and BrdU incorporation, respectively, after 24, 48, and 72 h. Gene expression of IL-6, TNF-α and AP was analyzed after 24 h.

### MTT assay

For model I, where cells were cultured in the presence of the scaffolds, the latter were removed from the plates just before 3-[4,5-dimethythiazol-2-yl]-2,5-diphenyltetrazolium bromide (MTT, 0.5 mg/ml, Sigma-Aldrich, St. Louis, MO, USA) was added. In model II, where the cells were cultured directly on the scaffold, MTT was added in each well and incubated for 2 h at 37 °C. Afterwards, formazan crystals, formed by the NAD(P)H-dependent oxidoreductases, were dissolved in dimethyl sulfoxide. Optical density was measured with a microplate reader (EL 808, Biotek Instruments, Winooski, VT, USA) and normalized to untreated cells.

### BrdU incorporation assay

For model I similarly to the MTT assay, scaffolds were removed from the culture plates before the BrdU incorporation assay was performed. For model II growth medium was removed, dental pulp fibroblasts were washed with PBS and serum-free media containing 5-bromo-2′-deoxyuridine (BrdU) was added to the cells for 2 h. BrdU incorporation was determined following the manufacturer’s instructions (Cell Proliferation ELISA, BrdU colorimetric kit from Roche; Basel, Switzerland) and normalized to untreated cells.

### Scanning electron microscopy

Surface structure topography of the scaffolds was examined by a scanning electron microscope at magnifications of × 500 at the Center of Microscopy and Image Analysis, University of Zurich (scanning electron microscopy (SEM); Carl Zeiss Supra 50 VP FESEM, Carl Zeiss). For this purpose, the samples were fixed for 24 h in 2.5% glutaraldehyde solution. Afterward, the scaffolds were rinsed with PBS and dehydrated in ascending concentrations of alcohol (50, 70, 80, and 90%) twice for 15 min. Finally, the scaffolds were immersed three times for 15 min in 94% and 60 min in 100% ethanol. Samples were then subjected to critical point drying (Bal-Tec CPD030), mounted on SEM mounts (Bal-Tec AG, Blazers, Liechtenstein), and were gold sputtered (Balzers SCD 030, Balzers Union, Balzers, Liechtenstein) for 60 s in an argon gas atmosphere at a target distance of 50 mm, at pressure of 5 Pascal (Pa) at 45 mA. SEM images were taken at a working distance of an acceleration voltage of 10 kV.

### Gene expression analysis

Twenty-four hours after stimulation, the scaffolds were either removed (Model I) or retained (Model II) for gene expression analysis. Total cellular RNA was isolated with the High Pure RNA Isolation Kit (Roche F. Hoffmann-La Roche, Basel, Switzerland). Reverse transcription (RT) was performed with Transcriptor Universal cDNA Master (Roche) and PCR was performed with the FastStart Universal SYBR Green Master (Roche) on a 7500 Real-Time PCR System (Applied Biosystems, Life Technologies Corporation, Carlsbad, CA, USA). Primers against the pro-inflammatory cytokines human IL-6 and TNF-*α* representing first reaction to an inflammation, as well as against AP as an early differentiation marker [20], and GAPDH, the house-keeping gene were designed in the online Universal ProbeLibrary Assay Design Center (Roche). The mRNA levels were calculated by normalizing to GAPDH using the ΔΔCt method.

### Statistical analysis

Data obtained by MTT and BrdU incorporation assays were reported as median, 25% percentile, 75% percentile, minimum and maximum of two independent experiments, each performed in duplicates. Differences between cells stimulated with the three scaffolds were tested using a non-parametric Kruskal-Wallis test followed by a post-hoc Mann-Whitney *U*-test. Furthermore differences between the two cell seeding methods and the three different stimulation periods were statistically analyzed (SPSS version 19.0, SPSS Inc., Chicago, IL, USA). Statistical significance was considered at *p* < 0.05. Data obtained by RT PCR, showing the differences in mRNA expression of target genes between cells stimulated with the different scaffolds and unstimulated cells were described by the mean and standard deviation.

## Results

**Fig. 1:**
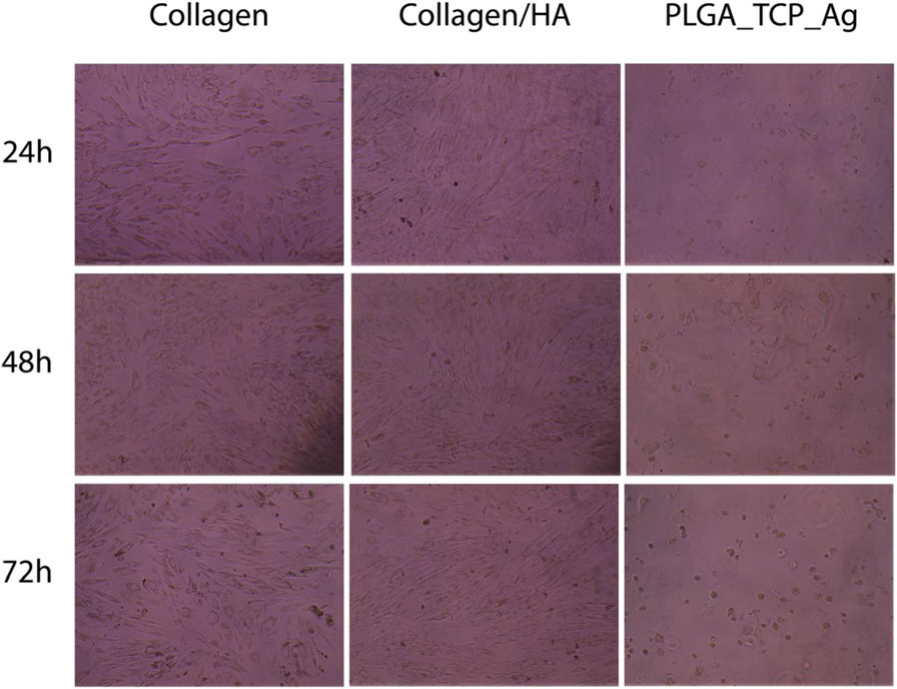
Morphology of dental pulp cells after stimulation with PLGA/Ag-TCP, Coll, and Coll-HA scaffolds for 24, 48, and 72 h, respectively

Dental pulp cells in all groups except for the Ag containing scaffold groups had a fibroblast like morphology without any signs of malaise and were attached over the whole period of 72 h (Fig. 1). In the Ag containing scaffold groups, cells were rounded up and most of them did not attach.

### PLGA/Ag-TCP scaffolds decrease formazan formation in human dental pulp-derived cells

**Fig. 2:**
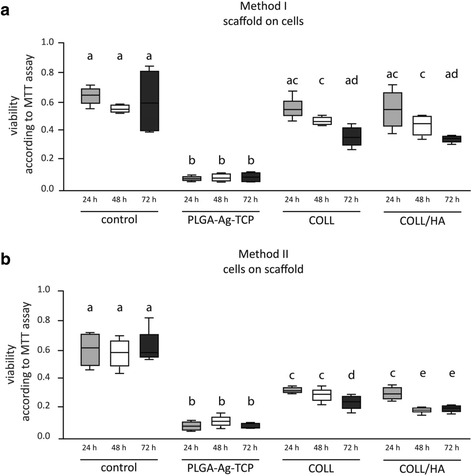
Viability of dental pulp fibroblasts measured by MTT assay after stimulation for 24h, 48h or 72h with PLGA/Ag-TCP, Coll, and Coll-HA scaffolds using two different seeding methods (**a**) the scaffolds were placed on the cells; (**b**) the cells were seeded on the scaffolds. Unstimulated cells acted as control. Different letter indicate statistically significant differences

Formazan formation as indicator for viability of dental pulp fibroblasts incubated with the scaffolds after 24, 48, and 72 h are shown in Fig. 2a and b.

When cells were cultured in the presence of the scaffolds (Model I) (Fig. 2a) the absolute viability values were higher than when the cells were cultured directly on the scaffolds (Model II) (Fig. 2b) while the overall trend with regard to the impact of the scaffold was the same. Formazan formation of cells incubated with PLGA/Ag-TCP was decreased after 24 h compared to the unstimulated control (w/o) (*p* < 0.05). In contrast, cells stimulated with Coll or Coll/HA using Model I did not show any differences compared to the unstimulated control (w/o) (*p* > 0.05) (Fig. 2a). After 48 and 72 h, all cells stimulated with scaffolds showed reduced levels of formazan formation, most prominently, when stimulation was performed with PLGA/Ag-TCP. When directly comparing the formazan formation of the cells that were stimulated with the three different scaffolds, substantial differences between the Ag containing scaffold and both collagen scaffolds were found. After 24, 48, and 72 h viability was significantly reduced when cells were stimulated with PLGA/Ag-TCP as compared to cells stimulated with Coll or Coll/HA (all *p* < 0.05). Comparing Coll and Coll/HA, no differences could be revealed at all times (all *p* > 0.05).

### PLGA/Ag-TCP scaffolds decrease BrdU incorporation in human dental pulp-derived cells

**Fig. 3:**
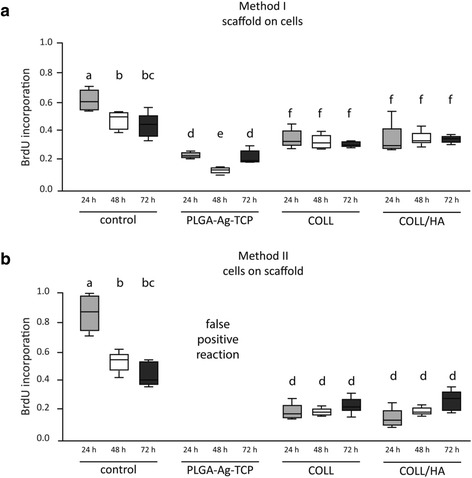
Proliferation of dental pulp fibroblasts measured by BrdU incorporation assay after stimulation for 24h, 48h or 72h with PLGA/Ag-TCP, Coll, and Coll-HA scaffolds using two different seeding methods (**a**) the scaffolds were placed on the cells; (**b**) the cells were seeded on the scaffolds). Unstimulated cells acted as control. Different letters indicate statistically significant differences

BrdU incorporation as indicator for proliferation of cells incubated with the scaffolds after 24, 48, and 72 h are shown in Fig. 3a and b.

In model II the PLGA/Ag-TCP scaffolds showed false positive reactions with the substrate solution TMB (3,3′,5,5′-Tetramethylbenzidine) of the BrdU assay. Nevertheless, the results of the BrdU assay from Coll and Coll-HA support the results in model I.

In model I, where cells were cultured in the presence of the scaffolds, incorporation of BrdU in dental pulp fibroblasts was decreased when cells were stimulated with PLGA/Ag-TCP or Coll compared to the unstimulated control (w/o) (*p* < 0.05). After 48 h, proliferation only decreased in the group with PLGA/Ag-TCP, whereas after 72 h, a decrease was again detectable in both groups (PLGA/Ag-TCP and Coll). A comparison of the proliferation rate of cells stimulated with the three different scaffolds showed that cells of the PLGA/Ag-TCP group achieved significantly lower values than both collagen groups (Coll and Coll/HA) after 24 and 48 h (all *p* < 0.05). Interestingly, after 72 h of stimulation, no significant differences between all three groups were detectable. Comparing Coll and Coll-HA, no differences could be revealed at all time points of stimulation (all *p* > 0.05; Fig. 3).

### Scanning electron microscopy

**Fig. 4:**
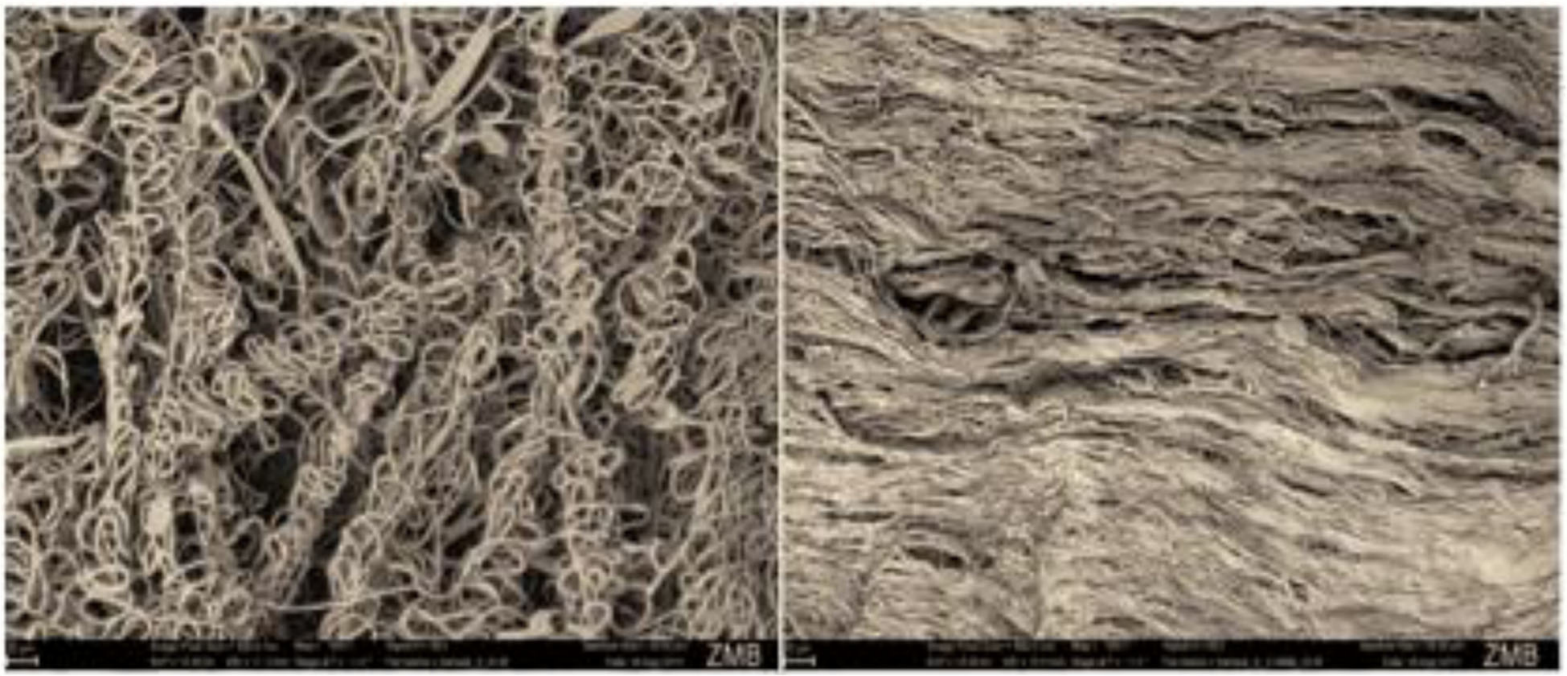
Scanning electron microscopy images of representative PLGA/Ag-TCP (*left*) and Coll scaffolds (*right*), respectively

Representative SEM demonstrated the typical woven appearance of the PLGA/Ag-TCP scaffold and the collagen structure of the resorbable scaffold. On a surface level, there was no difference between the untreated and hyaluronan treated scaffold surface, as the hyaluronan represents a material, which is very hydrophilic and thus spreads very well on the surface. Therefore, the surface of the scaffold was not affected. Furthermore, there is no chemical interaction between hyaluronan and scaffold, which might lead to a surface modification. Hyaluronan spreads on the scaffold and will act on cellular level due to its biochemical properties. The cross-linked hyaluronan will be absorbed on a very low level to its long molecular chain and due to the cross-linking (Fig. 4).

### PLGA/Ag-TCP scaffolds increase IL-6, TNF-α, and AP expression in human dental pulp-derived cells

**Fig. 5:**
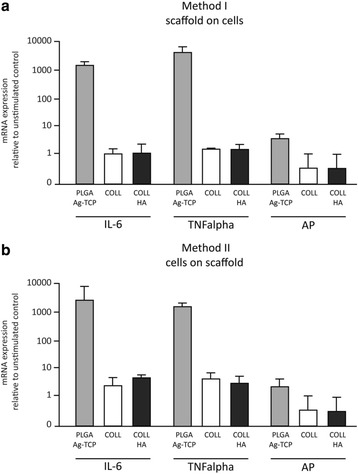
mRNA expression of dental pulp fibroblasts after stimulation with PLGA/Ag-TCP, Coll, and Coll-HA scaffolds using two different seeding methods (**a**) the scaffolds were placed on the cells; (**b**) the cells were seeded on the scaffolds. Unstimulated cells acted as control

Data for IL-6, TNF-*α* and AP gene expression are shown in Fig. 5a and b.

After stimulation of the dental pulp fibroblasts using cell seeding method I, i.e. scaffold on cells, expression of IL-6 and TNF-*α* substantially increased when PLGA/Ag-TCP was used, whereas the expression of IL-6 and TNF-*α* was compareable with no stimulation when Coll or Coll/HA were used. Regarding AP, cells stimulated with PLGA/Ag-TCP showed a slight increase in mRNA expression, while cells stimulated with Coll or Coll/HA showed a slightly decreased expression (Fig. 5a).

Gene expression values of the cell seeding method II, i.e. cells on scaffold, confirmed the results of cell seeding method I (Fig. 5b).

## Discussion

This proof of principle study assessed the cell viabilty and proliferation as well as the pro-inflammatory and metabolic capacity of human dental pulp cells in response to a novel silver-containing PLGA/TCP-nanofabric and compared it to collagen scaffold with and without hyaluronan acid (Coll and Coll-HA). It was hypothesized that PLGA/Ag-TCP induce minor reduction of cell viability and cell proliferation, which are comparable to the Coll and Coll-HA. Based on our results this hypothesis was rejected.

Considering also that the exposed dental pulp is a wound, the structure of the wound is important for the determination, differentiation, proliferation, survival, polarity and migration of cells [21]. Progresses in biomaterial science have significantly advanced, and scaffold materials have been tailored to mimic extracellular microenvironments of tissues or even organs to promote tissue regeneration under specific requirements in vivo [22]. In a recent study, Chen and co-workers tested the hypothesis that dental pulp/dentin complexes would contribute to the regeneration of tooth root and fabricated aligned PLGA/gelatin electrospun sheets (APES), treated dentin matrix (TDM) and native dental pulp extracellular matrix (DPEM), which were combined for periodontal or pulp regeneration, respectively [23]. Seeded with stem cells, the sandwich composites (APES/TDM/DPEM) generated tooth root-like tissues after being transplanted in porcine jaws for 12 weeks. In dental pulp/dentin complex-like tissues, columnar odontoblasts-like layer even arranged along the interface between newly formed predentin matrix and dental pulp-like tissues in which blood vessels could be found. This conceptual study showed that fibrous scaffolds are able to act as a potent control structure leading to a well-organized tissue formation of even original quality. However, under clinical conditions, such healing may be hampered by simple infections. Thus antimicrobial strategies have to be envisaged, which allow for appropriate cell homing.

Therefore, when thinking about the management of deep carious lesions and pulp affections in terms of capping procedures, bridging mechanisms are still warranted that encounter the effective disinfection and mineralization of the wound area. This study was based on this concept as well. A smart scaffold was designed, which combined antibacterial and bioactive properties, which seemed ideal for this indication. The nanofabric doped with silver has been shown to result in enhanced antimicrobial properties [13] when compared to tetracycline controls whilst being biocompatible in preclinical studies [14]. However, the immediate cell response within the limitations of the present in vitro investigation were shown to reduce cell viability and proliferation, while inducing a tremendous pro-inflammatory response and an increase in AP. This is in accordance with other findings with antimicrobial agents, which show evident cytotoxic effects in fibroblasts or osteoblasts [24, 25]. Long-term effects of the differentiation status of surviving cells, however, have not been investigated yet. These cells, despite being under stress and struggling for survival, can recover and form even mature osteoblasts even without external addition of growth factors and continue to deposit osteogenic cues into the newly formed extracellular matrix [26].

Summarizing the data of the present study, PLGA/Ag-TCP showed a toxic potential for dental pulp cells, while the collagen scaffolds with and without hyaluronan acid were well tolerated by the cells. This is particularly true for the 24 h data. However, after 72 h of stimulation a significant decrease in viability occurred in all groups. Whether this is of clinical significance could not be answered with the present in vitro testing method yet. Further investigations including a simulation of the physiological circulation might elucidate whether the cytotoxicity of PLGA/Ag-TCP is reversible or less dramatic, for instance by simple changing of the media.

The results of the BrdU assay may support the MTT findings. Over the entire study period of 72 h, the proliferation in the unstimulated control group substantially decreased. In the experimental groups, however, the proliferation remained at the same level during the entire investigative period, irrespective of the scaffold used. After 72 h, cells stimulated with PLGA/Ag-TCP even showed proliferation rates comparable to cells stimulated with Coll and Coll/HA. Two possible explanations could be drawn from these findings: either Ag is released from the scaffold and thereby affecting the assay, which might result in false positive results, or the cells enter a kind of “rescue state” and strongly attempt to proliferate. The latter assumption is strengthened by the results of the viability assay, and by the fact that the non-stimulated cells rather showed a decrease in the proliferation after 72 h, while all stimulated cells kept the proliferation rate at the same level over the entire period. Furthermore, the supernatant was removed directly before the assay. Together, it has to be stated that PLGA/Ag-TCP is toxic in this experimental set-up. Therefore, important questions still remain to be answered after this first proof of principle study. One critical question that needs to be further elucidated is whether the experimental outcome would have been the same when initial cell seeding would have been performed with fewer cells. In our results regarding viability and proliferation cells that were incubated with collagen or collagen with hyaluronan acid showed no dramatic changes over a period of 72 h. In contrast, with regard to cells incubated with PLGA/Ag-TCP we detected a decrease in the proliferation assay after 48 h, combined with an increase after 72 h. Our explanation of entering a “rescue state” might be indirectly checked by reducing the numbers of cells in the initial seeding in the other groups, where also an increase in proliferation might be seen. Our seeding density was 30,000 cells/cm^2^, which was based on previous in vitro studies [19]. Future studies will need to address the impact of the seeding density, especially since the literature on initial seeding density is divergent [27].

In this study, the MTT assay was used to assess cell cytotoxicity, which is a widely used approach [28]. In the MTT assay, the cellular activity with regard to the activity of NAD(P)H-dependent cellular oxidoreductase enzymes is certainly decreased under toxic conditions. However, the assay does not directly distinguish between the increase in cell numbers in the control vs. inhibition of cell division or reduction of cell numbers in the control group. Hence, enzyme release assays like the lactate dehydrogenase release assay or the Glyceraldehyde-3-Phosphate Dehydrogenase release assay where the enzymatic activity of soluble, cytosolic enzymes correlates with cell death would be a feasible approach [29]. However, the stable formazan formation and the reduction of BrdU incorporation over the 72 h observation period in the control group due to the chosen in vitro setup does not suggest that proliferation confounds our interpretation regarding the toxic effect. This is further supported by the morphological evaluation in the microscope. In addition, we have tried to determine the amount of silver in the medium, but there was either no silver ions released from the scaffolds or the concentration was below the detection limit of the applied method, i.e. in this case atomic absorption spectrometry.

Not surprisingly though, based on the toxic effects, the data of the RT-PCR showed an enhanced increase in inflammatory genes when cells were stimulated with PLGA/Ag-TCP for 24 h, thus the idea of entering a “rescue state” is supported. However, a slight increase in ALP was detected. In further studies it would be interesting to additionally evaluate the inflammatory response as well as a possible mineralization activity by using longer stimulation periods, maybe with osteogenic medium and to investigate these results also on a protein level. Furthermore, to reduce the toxicity of PLGA/Ag-TCP, while maintaining the known bactericidal effect and cause positive counter reaction of the cells. In a best-case scenario this might result in an increased migration of cells to the area of the exposed pulp, as well as an increased proliferation of the cells and tertiary dentin formation under a clinical situation. Besides, it would be interesting to know, if sorted stem cells from the dental pulp would show other reactions than the fibroblast like cells that were used in the experiments containing only 2–6% of cells that are positive for STRO-1 [30, 31].

A conceptual combination of hyaluronan acid and a silver scaffold, however, could also bear some benefits but also bear some problems as a recent article suggested [32]: Peritendinous adhesions, one of the common complications after tendon injury and subsequent surgery has led to the interesting development and investigation on a physical barrier between the injured site and the surrounding tissue using silver (Ag) nanoparticles embedded in electrospun hyaluronan acid (HA)/polycaprolactone (PCL) nanofibrous scaffolds (NFMs) (HA/PCL + Ag NFMs) to prevent peritendinous adhesions and bacterial infection after tendon surgery. This in vitro cell culture experiments revealed that HA/PCL + Ag NFMs exhibited the highest inhibition of fibroblast attachment and proliferation. Interestingly, in contrast to our findings, no significant cytotoxicity was observed, which was explained by the synergistic effect of Ag and HA and a lubrification effect. In the present study, this protective finding was not observed. Since a regeneration of tissues wants to be achieved, which includes the formation of the original tissues in dense contact to each other, the above mentioned contact inhibition seems not a desirable goal in this indication. Therefore, a combination of HA and Ag has to be carefully studied. Hyaluronan acid was chosen as potential candidate for improved wound healing in this screening study [18]. But the overall adjunctive beneficial effects of HA when on the collagen scaffolds in the present investigation were not that impressive within the limitations of this study. This might be due to the fact that the scaffolds were soaked in HA for a relatively short time of 1 min to stimulate the direct use in the clinical application for pulp capping procedures [33]. Maybe an incorporation of hyaluronan directly in the scaffolds or a pre-treating over a longer time period would change the cellular response, however, a longer pre-treatment is clinically not always possible, e.g. in the case of a direct pulp capping.

Regarding the two seeding methods investigated in this study, it became apparent that both methods are feasible since the outcomes are equal in all tested methods, which means that our second hypothesis was also rejected. Method I (scaffold on cells) appeared, however, better for this type of material testing. Firstly, this method better represents the clinical situation, since, for example at a direct pulp capping, the capping material has to be put on to the pulp tissue and not vice versa. Secondly, the direct comparison to the control group is more accurate in model I “scaffold on cells”, because always the same conditions are present. The other method (cells on scaffold) often misses a direct comparison group. Another advantage of the method I “scaffold on cells” is the easier implementation in the experimental procedure. The experiments are more accurate, faster and easier, thus less error-prone. In addition, considering the results of the proliferation assay, influence of the testing methodology by the scaffold can be excluded since the scaffold is removed directly before the investigations.

## Conclusion

Within the limitations of this in vitro study the following conclusions can be drawn:

PLGA/Ag-TCP initially decreased the viability and proliferation rate of human dental pulp cells while increasing the pro-inflammatory capacity and alkaline phosphatase expression. Whether surviving cells can enter a rescue state and therefore support the long-term survivability has to be determined in future studies. Furthermore a possible bactericidal effect has to be demonstrated.

Regarding the two different models it was obvious that Method I (scaffold on cells) was preferably using the presented study design.
